# Three-Dimensional Visualization of Mouse Endometrial Remodeling After Superovulation

**DOI:** 10.3389/fcell.2022.933852

**Published:** 2022-06-29

**Authors:** Yongcun Qu, Jia Zhang, Shanshan Guo, Liwen Zhang, Jingjing Qian, Xili Zhu, Enkui Duan, Ying Zhang

**Affiliations:** ^1^ Institute of Artificial Intelligence in Sports, Capital University of Physical Education and Sports, Beijing, China; ^2^ College of Life Sciences, Beijing Normal University, Beijing, China; ^3^ Institute of Zoology, Chinese Academy of Sciences, Beijing, China

**Keywords:** superovulation, uterine clearing, 3D imaging, uterine endometrial angiogenesis, uterine gland, steroid hormone

## Abstract

Uterine status determines pregnancy success. Although it is well known that superovulation operations can disrupt uterine function, our understanding of the morphological changes in the uterine endometrium at the three-dimensional (3D) level is limited. Here, combining the tissue clearing with 3D deep imaging, we reveal an increase in epithelial density and angiogenesis after ovarian stimulation, which is accompanied by a circulating surge in P4 levels. Using an ovariectomized mouse model, we further detected the separate regulatory effects of P4 and E2 on the uterine endometrium, with P4 promoting endothelial cell growth and E2 inducing epithelial proliferation. Additionally, we observed that the effects of E2 can be partially neutralized by P4, and vice versa. By analyzing the 3D uterine imaging, we discovered an interesting phenomenon in which the growing blood vessels closely surround the remodeling uterine epithelium, indicating a close relationship between angiogenesis and epithelial growth. These findings provide new insight into the uterine epithelial changes and angiogenesis at the 3D level, and explain a potential reason for endometrial changes due to the low implantation rate in patients undergoing clinic super-ovulation.

## Introduction

Although IVF (*in vitro* fertilization) has been widely used for over 40 years, a low implantation rate was found when fresh embryos were transferred after the superovulation cycle, especially in patients with ovarian hyperstimulation syndrome (Chen et al., 2016). Frozen embryo transfers can significantly increase the pregnancy rate (Chen et al., 2016; Shi et al., 2018), suggesting that the superovulation-induced abnormal uterine endometrial status is one of the main reasons for implantation failure.

In clinical and mouse-based researches, superovulation is a routine operation used to retrieve a large number of oocytes for pregnancy. However, this process usually induces an extensive ovulatory response and high levels of circulating steroid hormones. It is well known that progesterone (P4) and estrogen (E2) precisely regulate uterine functions, especially the changes in uterine blood vessels and the epithelium. For example, the induction of epithelial cell proliferation in mice is simply induced by E2 (O'Brien et al., 2006), but the regulatory effects of P4 and E2 on endometrial vasodilation and angiogenesis are more complex. It has been reported that E2 promotes uterine vascular permeability through its nuclear receptor ERα (Hyder et al., 2000), and stimulates VEGF and Flk1 expressions in the stromal bed (Ma et al., 2001), but profoundly attenuates uterine angiogenesis (Ma et al., 2001). P4 induces endothelial cell proliferation (Walter et al., 2005) and uterine angiogenesis but has little effect on vascular permeability (Ma et al., 2001). However, another study showed that E2 induces rapid proliferation of both the endothelial and stromal cells (Heryanto and Rogers, 2002), suggesting the angiogenic effect of E2. The differences between these results are not only due to the different mouse models used by the authors, but also due to the limitations of detection technology based on thin tissue sections, which can only provide local 2D information rather than 3D views. Moreover, although the adverse effects of superovulation and abnormal gene expression in the endometrium have been reported in the clinic (Haouzi et al., 2009), the specific changes in uterine glands and endometrial blood vessels remain unclear, especially at the 3D level.

Here, by combining the tissue clearing method and deep imaging, we detected 3D changes in uterine endometrial angiogenesis and epithelial proliferation in the superovulated mouse endometrium compared with the control endometrium, showing a decreased endometrial volume and increased epithelial and vascular density. For superovulation-induced circulating P4 levels rather than E2 levels in mice, we also used the ovariectomized mouse model to detect the regulation by P4 and E2 in the endometrium separately. Moreover, we also observed an interesting network between blood vessel growth and epithelial lining proliferation, indicating a potential interaction between them in the endometrium.

## Results

### Uterine 3D Imaging Revealed a Decreased Endometrial Volume Following Superovulation

**FIGURE 1:**
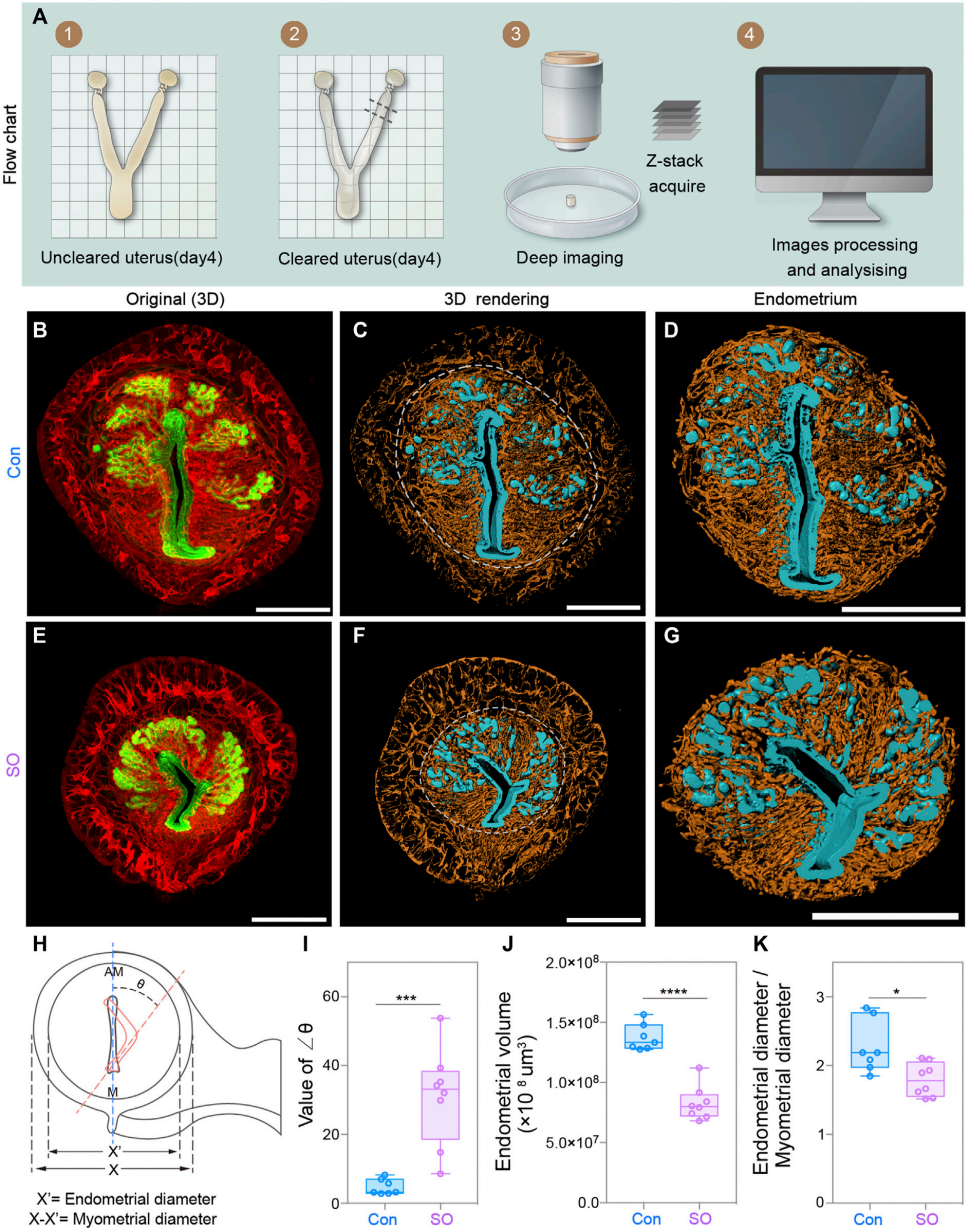
Uterine endometrial changes in the 3D level after superovulation. **(A)** Experimental flow chart. **(B)** 3D reconstruction of the control uterus on day 4. **(C–D)** 3D rendering of the control uterus **(C)** and uterine endometrium **(D)** on day 4. **(E)** 3D reconstruction of the superovulation uterus on day 4. **(F–G)** 3D rendering of the superovulation uterus **(F)** and uterine endometrium **(G)** on day 4. **(H)** Schematic diagram illustrating the luminal orientation within the uterus and the diameter of the endometrium and myometrium. The normal luminal axis is parallel to the uterine M-AM axis in the control uteri, while the abnormal axis (delineated by the orange dashed line) is deflected from the uterine axis in the superovulation uteri. The angles of deviation are indicated as ∠θ. **(I)** Comparing the value of the ∠θ in the control and superovulation groups. **(J)** Comparing the endometrial volume on day 4 in control and Superovulation groups. **(K)** Comparing the ratio of the endometrial diameter/myometrial diameter in the control and superovulation groups. Scale bar of **(B–G)** = 350 μm. The results are shown as means ± SEM. *p < 0.05, **p < 0.01, ***p < 0.001, ****p < 0.0001. n ≥ 7.

To detect morphological changes in the pre-implantation mouse uterus in response to exogenous gonadotropin, we established a uterine-specific whole-organ clearing method (Figure 1A), which was optimized from a previous work (Arora et al., 2016). Uteri from both control (Con) and superovulation (SO) mice at gestational day 4 (Day 4) were collected and cleared by using clearing methods, during which FLK1 (VEGF receptor 2) and CK (cytokeratin) were used to label vascular endothelial and epithelial cells of the uteri, respectively (Figures 1B–G). Combining confocal imaging and 3D reconstruction, we found that the uterus of SO group was much smaller than that of the Con group (Figures 1B,E). To further examine the endometrial changes under superovulation, we computationally isolated the uterine endometrium and myometrium separately, and the results showed that endometrial volume and the ratio for the endometrial/myometrial diameter were all significantly decreased following superovulation (Figures 1D,G,J,K), suggesting that superovulation might disrupt endometrial proliferation at Day 4.

More interestingly, we noticed that, unlike the vertical lumen along the mesometrial–antimesometrial (M–AM) axis in the uteri of the Con group, that of the SO group showed an aberrant fold of the lumen, and the luminal axis was deflected from the uterine axis (Figures 1H,I). The angles of deviation were indicated as ∠θ, which was >20° in the SO group but <10° in the Con group (Figure 1I). Vertical luminal closure on Day 4 is responsible for the correct embryo orientation during the implantation process. This defect of luminal folds might lead to many “pitfalls” for blastocysts, as a previous study reported (Zhang et al., 2014), and causes a potentially disoriented uterine–embryonic axis after implantation, which might lead to developmental failure of the embryo post-implantation.

### Superovulation Induced Epithelial Cell Proliferation and Endometrial Angiogenesis in Pre-implantation Uteri

**FIGURE 2:**
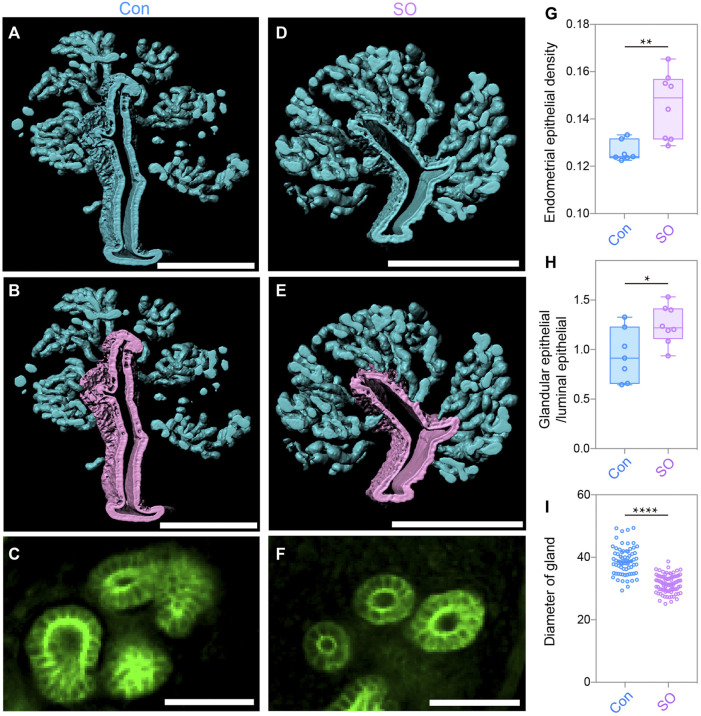
Uterine epithelial changes in the 3D level after superovulation. **(A)** 3D rendering of uterine epithelium of the control group. **(B)** 3D rendering of glandular epithelium (blue) and luminal epithelium (purple) of the control group. **(C)** Light-section of the uterine gland in the control group. **(D)** 3D-rendering of the uterine epithelium of the superovulation group. **(E)** 3D rendering of the glandular epithelium (blue) and the luminal epithelium (purple) of the superovulation group. **(F)** Light-section of the uterine gland in the superovulation group. **(G)** Comparing endometrial epithelial density in the control and superovulation groups. **(H)** Comparing the ratio of glandular epithelium/total epithelium in the control and superovulation groups. **(I)** Comparing the glandular diameter in the control and superovulation groups. Scale bar of **(A,B,D,E)** = 350 μm. Scale bar of **(C,F)** = 50 μm. The results are shown as means ± SEM. *p < 0.05, **p < 0.01, ***p < 0.001, ****p < 0.0001. n ≥ 7.

To further quantitatively analyze the endometrial changes, 3D surface of the epithelial signals was generated after separating the endometrium (Figures 2A,D). The statistical results showed an increase in the endometrial epithelial density after superovulation (Figure 2G). We then computationally isolated 3D surfaces of the luminal epithelium and glandular epithelium, and the 3D surfaces were evaluated. The purple signal indicates the luminal epithelium and the blue signal indicates the glandular epithelium (Figures 2B,E). Compared to the Con group, the SO group had more glandular epithelium, and the ratio for glandular epithelial volume/epithelial volume was increased by approximately 1.2 times (Figure 2H). However, when amplifying the glandular epithelium at the 2D level (Figures 2C,F), we found that the diameter of the gland was much smaller in the SO uteri than in the Con group (Figure 2I). All these results suggested that ovarian stimulation led to more epithelial cell proliferation but with narrower glands.

**FIGURE 3:**
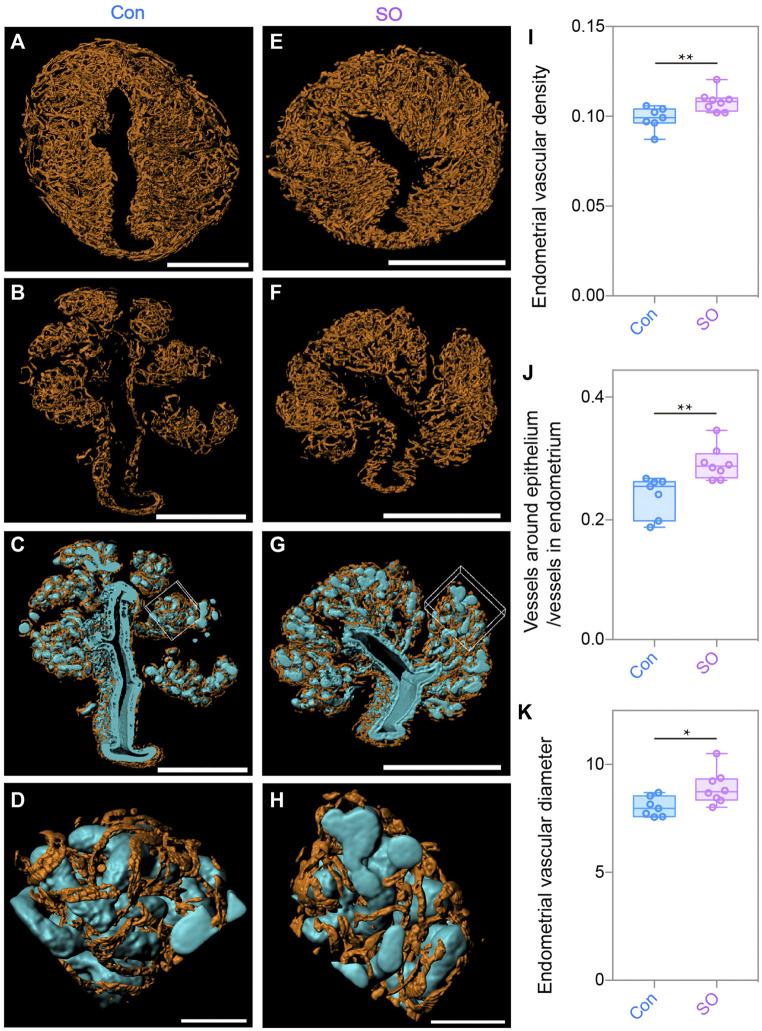
Uterine endometrial angiogenesis in the 3D level after superovulation. **(A)** 3D rendering of the endometrial blood vessels of the control group. **(B)** 3D rendering of the vessels around the epithelium in the control group. **(C)** Blood vessels closely surrounded the epithelium in the control group. **(D)** Magnified detail at the boxed area of **(C)**. **(E)** 3D rendering of endometrial blood vessels of the superovulation group. **(F)** 3D rendering of vessels around the epithelium in the superovulation group. **(G)** Blood vessels closely surrounding the epithelium in the superovulation group. **(H)** Magnified detail at the boxed area of **(G)**. **(I)** Comparing endometrial vascular density in the control and superovulation groups. **(J)** Comparing the ratio of the vessels around the epithelium/vessels in the endometrium in the control and superovulation groups. **(K)** Comparing the endometrial vascular diameter in the control and superovulation groups. Scale bar of **(A–C,E–H)** = 350 μm. Scale bar of **(D,H)** = 60 μm. The results are shown as means ± SEM. *p < 0.05, **p < 0.01, ***p < 0.001, ****p < 0.0001. n ≥ 7.

With the same strategy, we also generated the 3D surface of blood vessels (orange) to quantitatively calculate the changes in the endometrial angiogenesis. As shown in Figures 3A,E, we noticed that the endometrial vascular density and diameter of the SO group were a marginally increased compared with those of the Con group (Figures 3I,K), showing an abnormal angiogenesis pattern of the stimulated cycles. A previous study reported that excessive angiogenesis and an abnormal vascular diameter in the peri-implantation endometrium were positively correlated with the occurrence of a miscarriage (Plaisier, 2011), suggesting that the low implantation rate caused by superovulation could be related to an abnormal endometrial vascular growth.

During the analysis, we unexpectedly noticed that the glands were closely intertwined with growing blood vessels (Figures 3C,D,G,H), suggesting that there might be crosstalk of the proliferation between epithelial cells and endothelial cells, as the direction of epithelial cell growth might be regulated by angiogenesis, or vice versa. Considering the topological relationship between blood vessels and the epithelium, we assumed that the number of blood vessels surrounding the epithelium might also increase because a higher epithelial density was found in the superovulated group. To test this hypothesis, we computationally separated the blood vessels twisting around the epithelium to measure their volumes and found more growth of the blood vessels around the epithelium in the SO uteri (Figures 3B,F). Additionally, the ratio of vessels around the epithelium to endometrial vessels was higher than that in Con group’s uteri (Figure 3J). Taken together, the 3D-imaging results indicate that superovulation induced uterine epithelial cell proliferation and endometrial angiogenesis, which impaired the pre-implantation uterine milieu at the pre-implantation stage.

### Superovulation Increased Circulating Progesterone Levels at the Early Stage of Pregnancy

**FIGURE 4:**
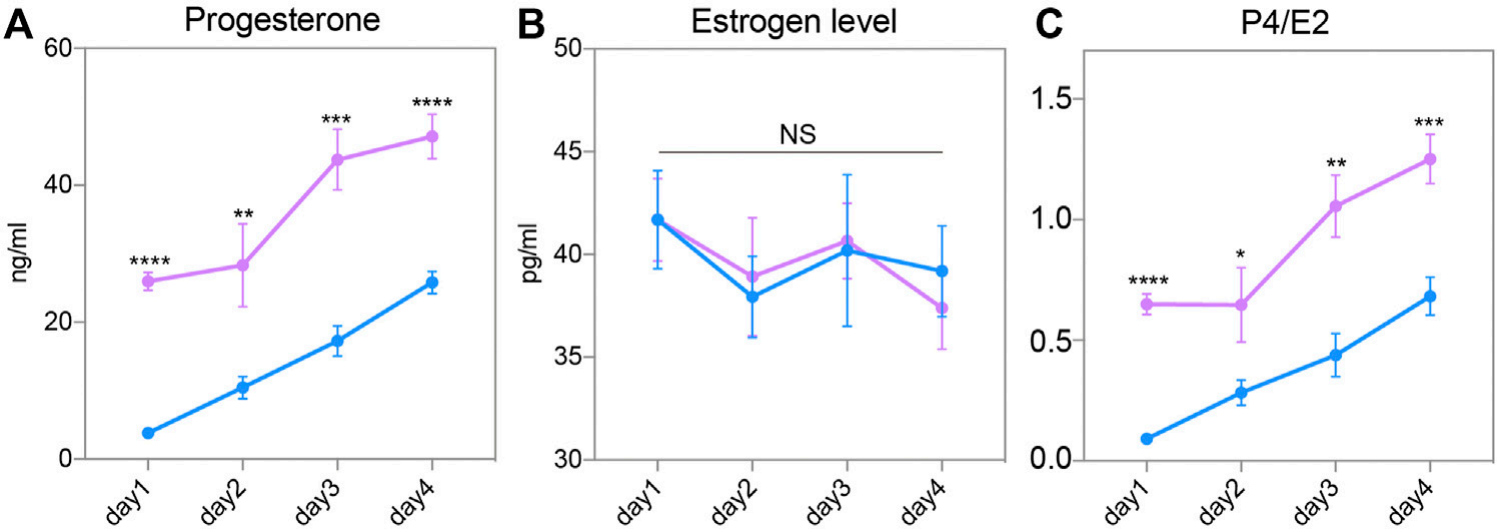
Steroid hormone in response to superovulation. **(A)** Circulating progesterone (P4) levels in the control (blue) and superovulation (magenta) groups. **(B)** Circulating estrogen (E2) levels in the control (blue) and superovulation (magenta) groups. **(C)** The ratio of P4/E2 in control (blue) and superovulation (magenta) groups. The results are shown as means ± SEM. *p < 0.05, **p < 0.01, ***p < 0.001, ****p < 0.0001. n ≥ 5.

To explore the mechanism of endometrial changes, we measured the circulating P4 and E2 levels of female mice after routine superovulation. After successful mating with wild-type male mice, the serum of pregnant female mice were collected from Day 1 to Day 4. The circulating P4 level continually increased from Day 1 to Day 4 in both groups. However, serum P4 was significantly higher in the SO group than in the Con group on these 4 days (Figure 4A). E2 showed relatively gentle changes in the pre-implantation stage, but there was no difference between the Con and SO groups (Figure 4B). Because the ratio of P4 to E2 is a key regulator of implantation, we also checked the changes in this ratio, and found a higher ratio in the SO group (Figure 4C), indicating that the superovulation-induced serum P4 level might be the cause of the endometrial changes.

### Abnormal Endometrial Alterations Following Superovulation Were Mainly Regulated by Increased Progesterone

**FIGURE 5:**
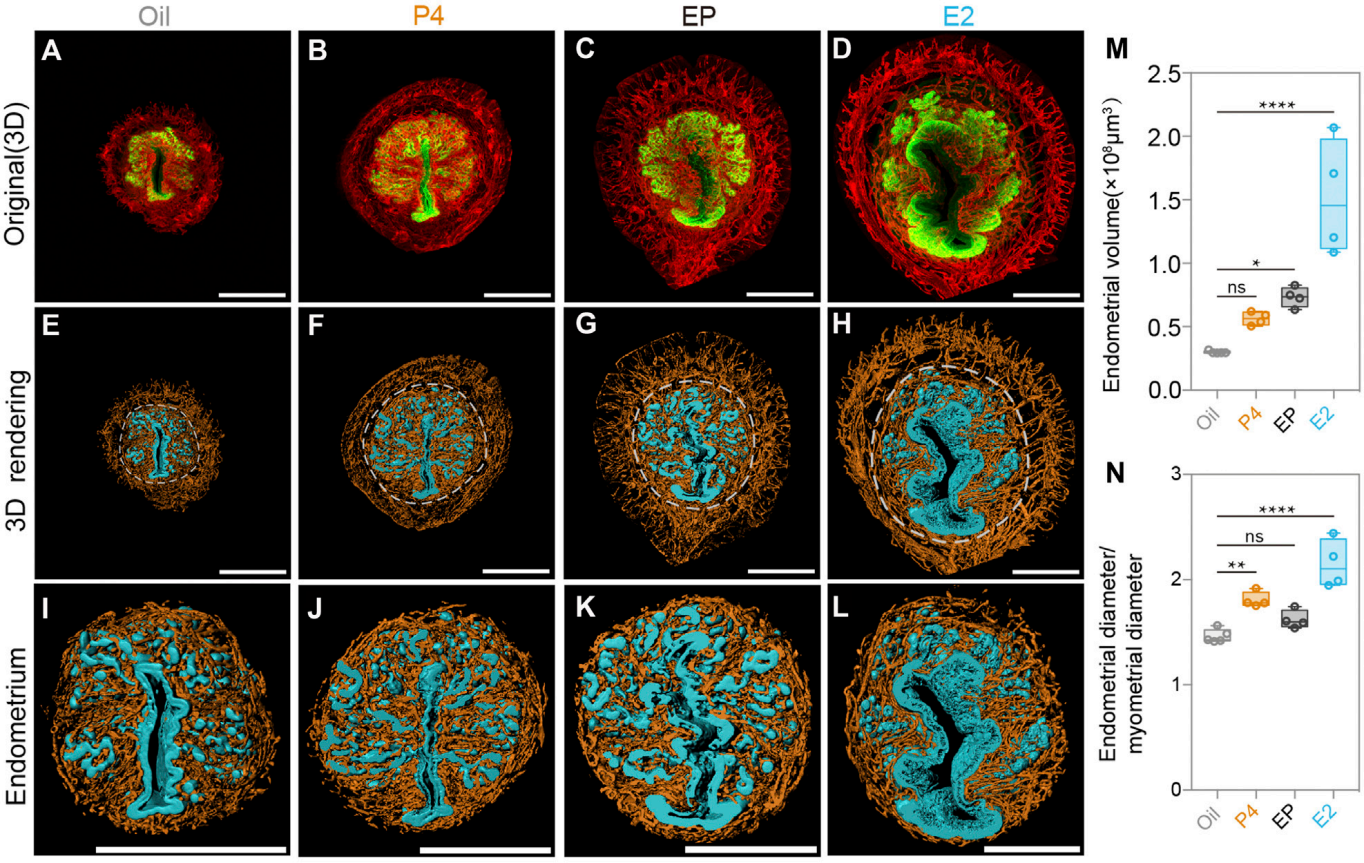
Uterine endometrial changes in the 3D level after steroid hormone treatment in ovariectomized mice. **(A–D)** 3D reconstruction of the uterus after the oil-treated group **(A)**, P4-treated group **(B)**, E2 plus P4 (EP)-treated group **(C)**, and E2-treated group **(D)**. **(E–H)** 3D rendering of the uterus after the oil-treated group **(E)**, P4-treated group **(F)**, E2 plus P4 (EP) treated group **(G)**, and E2-treated group **(H)**. **(I–L)** 3D rendering of uterine endometrial after the oil-treated group **(I)**, P4-treated group **(J)**, E2 plus P4 (EP)-treated group **(K)**, and E2-treated group **(L)**. **(M)** Comparing endometrial volume with different steroid hormone treatments. **(N)** Comparing the ratio of the endometrial diameter/myometrial diameter with different steroid hormone treatments. Scale bar of **(A–L)** = 350 μm. The results are shown as means ± SEM. Different letters represent significant differences (*p* < 0.05). *p < 0.05, **p < 0.01, ***p < 0.001, ****p < 0.0001. n ≥ 4.

To further explore the regulatory role of P4 in endometrial changes at the 3D level, we established an ovariectomized mouse model (OV), and found that the ovariectomized uteri were much smaller than the normal uteri due to the lack of hormones, as previously reported. We treated the OV mice with sesame oil, P4, E2, and P4 plus E2 (EP) separately for 72 h. Then, the dissected uteri were collected and 3D imaging analyses as those for the SO and Con groups were performed. As shown in Figures 5A–L, the P4-, E2-, and EP-treatments all induced the proliferation of the uterine endometrium, which is consistent with previous reports. We also observed an increase in endometrial volume and the endometrium/myometrium ratio (Figures 5M,N). Among the hormone-treated groups, we noticed that although E2 treatment significantly promoted endometrial proliferation, the addition of P4 to E2 could partially counteract the E2 stimulatory effects (Figures 5M,N). This observation could explain the phenotype we found in the SO group that with the existence of E2, surged P4 decreased the endometrial volume during the pre-implantation stage.

**FIGURE 6:**
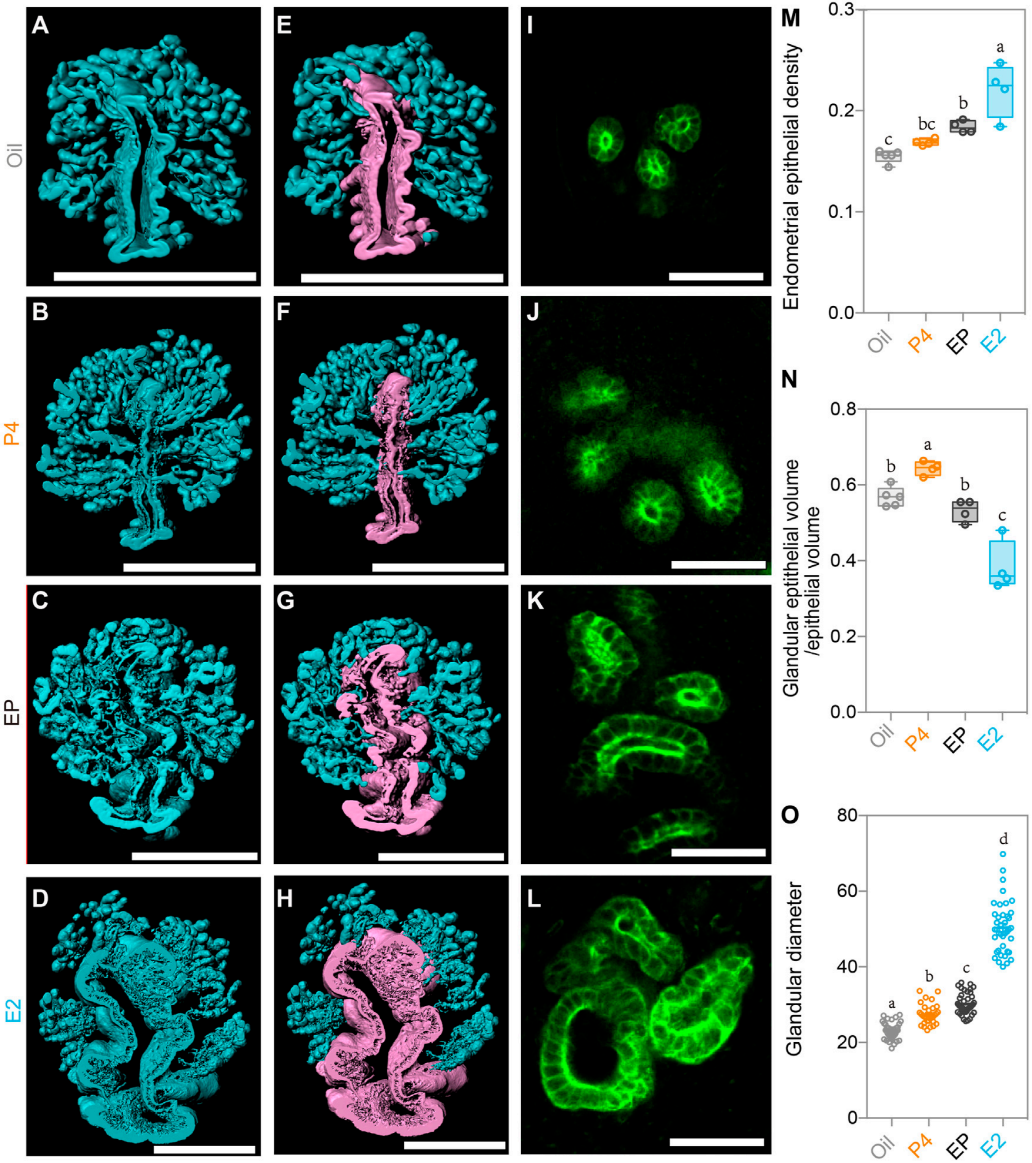
Uterine epithelial alteration in the 3D level after steroid hormone treatment. **(A–D)** 3D rendering of the uterine epithelium of the oil-treated group **(A)**, P4- treated group **(B)**, E2 plus P4 (EP)-treated group **(C)**, and E2-treated group **(D)**. **(E–H)** 3D rendering of the glandular epithelium (blue) and luminal epithelium (purple) of the oil-treated group **(E)**, P4-treated group **(F)**, E2 plus P4 (EP)-treated group **(G)**, and E2-treated group **(H)**. **(I–L)** light-section of the uterine gland in the oil-treated group **(I)**, P4-treated group **(J)**, EP-treated group **(K)**, and E2-treated group **(L)**. **(M)** Comparing the endometrial epithelial density with different steroid hormone treatments. **(N)** Comparing the ratio of glandular epithelium/total epithelium with different steroid hormone treatments. **(O)** Comparing the glandular diameter with different steroid hormone treatments. Scale bar of **(A–H)** = 350 μm. Scale bar of **(I–L)** = 50 μm. The results are shown as means ± SEM. Different letters represent significant differences (*p* < 0.05). *p < 0.05, **p < 0.01, ***p < 0.001, ****p < 0.0001. n ≥ 4.

We next detected changes in the endometrial glands. Although the E2 treatment induced endometrial gland growth as the endometrial epithelial density increased significantly, the administration of P4 plus E2 remarkably reduced the endometrial epithelial density (Figure 6M), indicating the inverse effect of P4 compared to E2. On the other hand, after isolating luminal and glandular epithelia separately as we did previously (Figures 6A–H), we found different effects between P4 and E2 on the glandular and luminal epithelia. The ratio of glandular epithelium/luminal epithelium was highest in the P4 group and lowest in the E2 group (Figure 6N), and P4 plus E2 neutralized the effect of administration of these two hormones alone (Figure 6N). A similar phenomenon was also found when analyzing the glandular diameter. E2 treatment alone induced the largest glandular diameter, but if treated with P4 plus E2, the glandular diameter was markedly decreased (Figures 6I–L,O). All these results suggested opposing the effects by P4 and E2 on epithelial cell growth, which can help us to understand the epithelial changes in the SO groups. In the Con group, the P4 level gradually increased from Day 1 to Day 4, while the E2 level remained stable during the pre-implantation stage. However, in the SO group, excess P4 was induced, while the E2 level was unchanged, which promoted glandular epithelial growth and resulted in an increase in the endometrial epithelial density and the glandular epithelium/luminal epithelium ratio.

**FIGURE 7:**
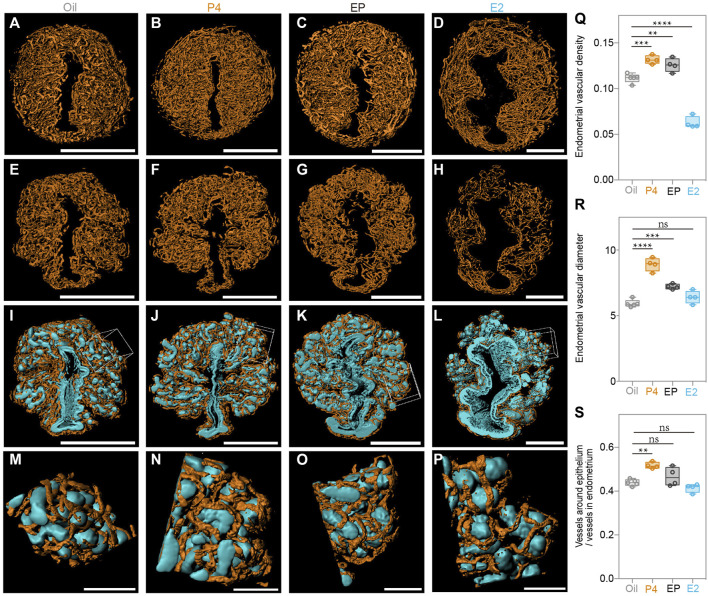
Uterine endometrial angiogenesis in the 3D level after steroid hormone treatment. **(A–D)** 3D rendering of endometrial blood vessels in the oil-treated group **(A)**, P4-treated group **(B)**, E2 plus P4 (EP)-treated group **(C)**, and E2-treated group **(D)**. **(E–H)** 3D rendering of the vessels around the epithelium in the oil-treated group **(E)**, P4-treated group **(F)**, E2 plus P4 (EP)-treated group **(G)**, and E2 treated group **(H)**. **(I–L)** Blood vessels closely surrounding the epithelium in the oil-treated group **(I)**, P4-treated group **(J)**, E2 plus P4 (EP)-treated group **(K)** and E2-treated group **(L)**. **(M)** Magnified details at the boxed area of **(I)**. **(N)** Magnified details at the boxed area of **(N)**. **(O)** Magnified details at the boxed area of **(K)**. **(P)** Magnified details at the boxed area of **(L)**. **(Q)** Comparing the endometrial vascular density with different steroid hormone treatments. **(R)** Comparing the ratio of vessels around the epithelium/vessels in the endometrium with different steroid hormone treatments. **(S)** Comparing the endometrial vascular diameter with different steroid hormone treatments. Scale bar of **(A–L)** = 350 μm. Scale bar of **(M–P)** = 70 μm The results are shown as means ± SEM. Different letters represent significant differences (*p* < 0.05). *p < 0.05, **p < 0.01, ***p < 0.001, ****p < 0.0001. n ≥ 4.

Finally, we generated a 3D surface of the blood vessels in the endometrium of all the groups (Figures 7A–D), and observed that, compared to the oil-treated group, P4 significantly promoted endometrial blood vessel growth, while E2 inhibited this process. The endometrial vascular density and diameter were highest in the P4-treated group but lowest in the E2-treated group (Figures 7Q,R). It was not surprising to find that the P4 effects were attenuated by E2, and the endometrial vascular density and diameter of the EP-treated group were all between those of the P4 and E2 groups. Additionally, the ratio of the vessels around the epithelium/vessels in the endometrium was increased by P4 treatment (Figures 7E–H,S), suggesting that endometrial angiogenesis after superovulation could be regulated mainly by high P4 levels. In addition, blood vessels closely surrounded the epithelium in all groups as we found in the SO uteri (Figures 7I–P), suggesting that this topological relationship could play important roles in the endometrial function.

## Discussion

In the present study, we established a uterine-specific whole-organ clearing method and examined the 3D morphological changes in the superovulation mouse endometrium. We found a routine superovulation-induced epithelial cell proliferation and endometrial angiogenesis in the pre-implantation uteri, following a remarkable surge in circulating progesterone. We also revealed an essential finding that the growing blood vessels closely surrounded the remodeling uterine epithelium, which is the first morphological evidence of the close relationship between epithelial proliferation and angiogenesis at the 3D level.

Superovulation has been reported to cause many pregnancy disorders, among which defects in uterine receptivity are one of the main causes. Although most previous studies have focused on the molecular changes of uterine cells that regulate the receptivity establishment, the morphological differences of the uteri were more obvious to help us judge the uterine status. Here, by employing 3D imaging and quantitative analysis, we illustrated the changes in the endometrial glands and blood vessels following superovulation. It has been proved that uterine glands are essential for reproduction. The uterine gland knockout (UGKO) sheep and mice all showed infertility, and both had a defect in embryo implantation (Filant and Spencer, 2014). Moreover, the impaired uterine glands also affected glandular secretions, which are an important information mediator between the floating embryo and maternal uterus, and led to incorrect communication (Zhang et al., 2017). We observed the abnormal proliferation of the uterine gland after superovulation, indicating that glandular secretion might be changed. On the other hand, a previous study showed that the mouse uterus should show normal luminal closure along the M-AM direction on Day 4, to ensure that the embryo implants are in the right site, and if these processes are destroyed, aberrant embryo implantation will lead to a progressively disoriented embryonic–uterine axis and miscarriage (Zhang et al., 2014). Our findings in the superovulation mice showed an incorrect luminal structure in the SO uteri, suggesting potential adverse effects for embryo implantation.

Angiogenesis is another important event that regulates uterine receptivity. After the uterine arteries perfuse and branch into the myometrial layer, small vessels arrive at the endometrium. As early as 1940, two different endometrial vessels were described, including the straight arterioles that are in the basal layer and spiral arterioles that are in the functional layer. Among them, spiral arterioles have been found to be sensitive to progesterone (Karizbodagh et al., 2017). Here, we found that superovulation induced the endometrial angiogenesis, which is consistent with previous studies (Rashidi et al., 2017). Interestingly, we first discovered that the growing blood vessels closely surrounded the remodeling uterine glands at the 3D level, indicating the interaction between the epithelial cell proliferation and endothelial cell growth. Considering that the majority of endometrial VEGF is produced by glands (Gargett et al., 1999), there might be a higher expression of VEGF in the uterine gland proliferation site, which might direct the angiogenesis, but this hypothesis needs more study.

Progesterone and estrogen are the main regulators of the uterine status and the process of embryo implantation. It is well established that the function of these two hormones occurs primarily through their receptors via the activation of signaling cascades and adjustments to gene expression (Marquardt et al., 2019). During the pre-implantation stage, progesterone plays an essential role in establishing uterine receptivity, and supplementation with progesterone may extend uterine receptivity for implantation (Song et al., 2007). However, additional evidence has demonstrated that a high concentration of progesterone might impair uterine receptivity and implantation (Liang et al., 2018) and that an aberrant progesterone/estrogen ratio is also harmful to early pregnancy (Maclin et al., 1990), indicating that the concentration of progesterone should be in a suitable range to support implantation. Here, we discovered that a routine superovulation operation increases progesterone and the progesterone/estrogen ratio in mice, which was consistent with previous studies (Akinlosotu and Wilder, 1993). Considering the neutralization effects between E2 and P4, the adjustment of the serum P4/E2 ratio might be helpful in clinical treatments under a similar situation.

On the other hand, our study also has several limitations. Although superovulation-induced epithelial and vascular abnormalities at the 3D level, the adverse effects of these changes on implantation and embryo development in later pregnancy are still unclear. Moreover, in our study, we only measured the serum progesterone and estrogen levels in pregnant mice after ovarian stimulation and did not include other steroid hormones. It has been reported that superovulation could robustly elevate the serum androgen levels, which could also promote epithelial proliferation via androgen receptors in the epithelium, suggesting that endometrial structural alterations may not only be due to a higher progesterone level. Furthermore, it is impossible to reveal the continuous changes of the endometrium in the 3D level for the limitations of tissue clearing methods, which have to use the fixed samples. It is worth expecting the real-time 3D imaging that helps us to have a direct view of the process of embryo implantation.

In summary, we used the uterine 3D reconstruction technology to discover that the superovulation operation increased the circulating progesterone and induced an abnormal epithelial cell proliferation and angiogenesis, which provides new insight for the evaluation of the impaired endometrial receptivity caused by ovarian stimulation at the 3D level, and also might provide a potential clinical index for predicting the outcome of IVF in the future.

## Materials and Methods

### Mice

Eight-week-old adult CD1 mice were purchased from Charles River Laboratories China Inc. The mice were maintained with a 12:12-h light–dark cycle, and the health status is specific pathogen-free according to the Animal Care and Use Committee of Institute of Zoology, Chinese Academy of Sciences.

### Superovulation Procedure and Conventional Mating

Virgin adult CD1 female mice were randomly divided into two groups; superovulation group of mice (SO) were injected with 7.5 IU PMSG (Pregnant Mare Serum Gonadotropin), following 7.5 IU HCG (Human Chorionic Gonadotrophin) 48 h later, and the control group (Con) were injected with a saline solution. Virgin adult CD1 female mice of the SO and Con groups were separately mated one-to-one with males overnight and the morning (08:00 a.m.) finding of the vaginal plug was designated as day 1 of pregnancy and then pregnant mice were sacrificed on day 4 (09:00 a.m.) of pregnancy.

### Steroid Hormone Injection

Ovariectomized mice were respectively injected with sesame oil (0.1 ml/mouse), estradiol-17β (E2) (100 ng/mouse), P4 (2 mg/mouse), and E2 plus P4 at 9:00 a.m. every day, for 3 days, and then were sacrificed, and their uteri were collected 72 h after the first injection.

### Measurement of Serum Hormone Levels

Blood samples of superovulation female pregnant mice and control female pregnant mice on day1–day4 were separately collected by eyeball extirpating under diethyl ether anesthesia. Blood samples were centrifuged at 3,000 rpm for 10 min at 4°C, and the serum was collected for estrogen and progesterone concentration detection using the electro-chemiluminescent immunoassay method.

### Whole Mount Immunostaining

Whole mount immunostaining and tissue clearing methods were optimized as previously reported (Arora et al., 2016). Briefly, 1) all the female mice were sacrificed by cervical dislocation and the uteri were placed into fixative (DMSO:Methanol, 1:4) at −20°C overnight. 2) The fixed uteri were transferred to a 1:1, Methanol:PBT (PBS +1% Triton) solution at room temperature rocking on a nutator for 20 min 3) The uteri were transferred to 100% PBT at room temperature rocking on a nutator for 20 min 4) The uteri were blocked in PBSMT (PBS+1%Triton+2%powdered milk) at room temperature rocking on a nutator for 2 h 5) The uteri were subjected to immunostaining using primary antibodies at a dilution of (1:100) in PBSMT and incubated for 5 nights at 4°C on nutator. 6) After 5 days of incubation, the uteri were washed 7 times with PBSMT for 35 min on a nutator at room temperature. 7) The uteri were subjected to immunostaining using secondary antibodies at a dilution of (1:100) in PBSMT and incubated for 3 nights at 4°C on a nutator, and all the samples were covered with an aluminum foil to keep them in the dark. 8) After this step, the uteri were washed 7 times with PBSMT for 35 min on a nutator at room temperature.

### Tissue Clearing Procedure

1) After immunofluorescence staining, the uteri were washed 7 times with PBSMT for 35 min on a nutator at room temperature. 2) The uteri were then transferred to 1:1, Methanol:PBT (PBS +1% Triton) solution for 5 min on a nutator at room temperature. 3) The uteri were transferred to a 100% methanol solution for 15 min each time on a nutator at room temperature, twice in total. 4) The uteri were cross-cut into 4 mm tissues and transferred to a 3% H_2_O_2_ solution (H_2_O_2_ diluted in methanol) and incubated 12 h on the nutator at 4°C. 5) All uterine tissues were then transferred to 100% methanol for 30 min at room temperature while rocking on a nutator. 6) All uterine tissues were put on filter papers to absorb the methanol completely and then immersed in BABB (Benzyl alcohol: Benzyl Benzoate, 1:2) until the tissues were transparent.

### Image Data Obtaining and Analysis

Clarified uteri tissues were put upright on glass bottom culture dishes (NEST) and imaged with a Zeiss LSM 780 confocal microscope using 25X lenses. Tile scans were stitched using Zeiss software. The imaging data analysis, mainly performed with the Imaris software (8.4.1, Bitplane), and the Concrete analysis were as follows: The GFP signal and RFP signal were used to generate a surface of the epithelium and blood vessels separately for quantitative analysis. The surface gain size and diameter of the largest sphere were fit into the object, and the threshold value setting was consistent with the image signal. Under the 3D surface module, the uterine endometrium was circled according to uterine autofluorescence, and the endometrial volume, epithelial volume, and vascular volume were also determined. The separation of the glandular epithelium and luminal epithelium, as well as the splitting of vessels around the epithelium was also under 3D surface module, whereas the diameter of vessels was detected under filament modules. In addition to the aforementioned analysis, the diameter of the uterine myometrium, endometrium ,and diameter of the gland, as well as the angle between the uterine vertical axis (AM–M axis) and the uterine luminal axis were determined by using ImageJ software.

### Data Statistics

Quantitative data were subjected to analysis by using the two-tailed unpaired Student’s t-test (2 groups), or an ordinary one-way ANOVA (>2 groups) with multiple comparisons test. The results were analyzed with GraphPad Prism 7 and presented as mean ±; statistical significance was set as *p* < 0.05.

## Data Availability

The original contributions presented in the study are included in the article/Supplementary Material; further inquiries can be directed to the corresponding author.
